# Associations of brain‐derived neurotrophic factor rs6265 polymorphism and cognitive function in breast cancer survivors from a cross‐sectional study

**DOI:** 10.1002/cam4.6975

**Published:** 2024-02-04

**Authors:** Taichi Goto, Leorey N. Saligan, Xiaobai Li, Lichen Xiang, Catherine Kwiat, Christopher Nguyen, Adele Crouch, Diane Von Ah

**Affiliations:** ^1^ Symptoms Biology Unit, Division of Intramural Research National Institute of Nursing Research, National Institutes of Health Bethesda Maryland USA; ^2^ Department of Biostatistics National Institutes of Health Clinical Center Bethesda Maryland USA; ^3^ Indiana University School of Nursing Indianapolis Indiana USA; ^4^ The Ohio State University College of Nursing Columbus Ohio USA

**Keywords:** BDNF, cancer‐related cognitive impairment, Val66Met, working memory and executive function

## Abstract

**Background:**

Breast cancer survivors (BCS) often complain of cancer‐related cognitive impairment (CRCI) during and even months after completing primary cancer treatments, particularly chemotherapy. The etiology of CRCI is unknown, but associations of CRCI with germline genetic polymorphisms have been reported, including Brain‐Derived Neurotrophic Factor (BDNF) rs6265 polymorphism. The current study investigated the associations of specific BDNF rs6265 with CRCI.

**Methods:**

Cancer‐related cognitive impairment was assessed using subjective reports of cognitive symptoms (the version 1.0, 8‐item short‐forms of the Patient‐Reported Outcomes Measurement Information System®) and computerized objective cognitive function scores (CANTAB®). BDNF rs6265 genotypes were determined from buccal swabs. The associations of specific BDNF rs6265 with CRCI were examined by either one‐way analysis of variance or the Kruskal–Wallis test followed by post hoc tests and rank‐based regression analysis.

**Results:**

We examined 356 female BCS. The mean (SD) age was 55.6 (9.8) years old, the median (IQR) years since cancer diagnosis were 4.0 (6.0), and 331 (92.7%) were self‐described as White. BCS carrying the Met/Met genotype showed poorer results on ‘visual episodic memory and new learning’ and ‘spatial working memory and executive function.’ This relationship was observed regardless of prior chemotherapy.

**Conclusion:**

Our findings suggest that carrying the BDNF rs6265 Met/Met genotype increases the risk for CRCI in BCS. These results are foundational in nature and provide important information to identify mechanisms underpinning CRCI.

## 1 INTRODUCTION

Cancer‐related cognitive impairment (CRCI) often affects breast cancer survivors (BCS) during and even after their primary cancer treatments.^1^, ^2^, ^3^, ^4^ Cancer survivors including BCS often express concerns or problems with their memory, concentration, and problem‐solving.^5^ Objective assessments have identified deficits in episodic memory and learning, sustained attention, working memory, and executive function.^6^ CRCI is identified in 20%–35% of BCS post‐treatment^7^ and has been shown to have a significant and detrimental impact on work ability, everyday function, and quality of life.^5^, ^8^ Despite the common and potentially debilitating symptoms of CRCI, the underlying mechanism of CRCI is still understudied. Unveiling the mechanisms underpinning CRCI can lead to the development of new therapeutic strategies for this functional burden.

Many factors have been implicated to predispose BCS to CRCI.^9^ Recent studies have examined the role of single nucleotide polymorphisms (SNP) and their association with CRCI.^10^ One review explored the relationships of subjective reports with objective measures of CRCI with germline genetic polymorphisms in women with early‐stage breast cancer while receiving and after completing their primary treatments.^10^ One particular gene of interest they found was the brain‐derived neurotrophic factor (*BDNF*), which is a neurotrophin with important roles in neuronal survival and differentiation, as well as the regulation of synapses.^11^, ^12^ This specific *BDNF* (rs6265) polymorphism that has been associated with several cognitive symptoms involves the replacement of methionine with valine (Val66Met). These symptoms included a decline in episodic memory function, decreased hippocampal activity in learning tasks, and increased susceptibility to anxiety and depression.^13^, ^14^, ^15^, ^16^ Functionally, it has been revealed that the Val66Met substitution causes a decrease in activity‐dependent release of BDNF,^13^ negatively altering hippocampal function and thus a decline in episodic memory. While there is evidence showing a decline in cognitive skills, such as specific types of memory and perceptual speed associated with aging, in patients with the Met allele,^17^, ^18^ other studies have reported that older adults with Met66 have higher cognitive ability, decreased Alzheimer's disease risk and improved episodic memory.^19^, ^20^, ^21^, ^22^ These contradictory effects and age‐dependent consequences of the *BDNF* rs6265 polymorphism among cancer survivors were also documented in another systematic review.^23^ Therefore, further investigations addressing some of the unanswered questions contribute to the existing body of knowledge in this field.

The current study explored the associations of *BDNF* rs6265 polymorphism with specific cognitive function domains among female BCS. We hypothesized that BCS carrying the *BDNF* rs6265 Met/Met genotype would have decreased cognitive function domain scores than BCS carrying the Val/Val genotype.

## 2 METHODS

### 2.1 Study sample

The current sub‐analysis is part of a parent study examining factors associated with CRCI in cancer survivors (ClinicalTrials.gov Identifier: NCT04611620). This sub‐analysis focused on initially exploring the relationships of *BDNF* rs6265 polymorphism with CRCI among female BCS. The recruitment methods and eligibility criteria of the parent study are described in our previous paper.^24^ Briefly, we enrolled BCS if they were ≥21 years old, were at ≥6 months after completing adjuvant/neo‐adjuvant therapies for breast or colorectal cancer (current Aromatase Inhibitors or Tamoxifen use at the time of enrollment was allowed), and had self‐reported CRCI. In this analysis, only female BCS with early‐stage BC (I–III) were included. Before study entry, online informed consent was obtained through a secure HIPAA‐compliant REDCap^®^ form. The study was approved by a large comprehensive cancer center in the Midwest and the University Institutional Review Board.

### 2.2 Patient‐reported outcomes

Participant characteristics, including demographic information and medical/treatment information, were obtained using an investigator‐initiated sociodemographic form.^24^ The version 1.0, 8‐item short‐forms of the Patient‐Reported Outcomes Measurement Information System (PROMIS)^®^ Cognitive Abilities and Cognitive Concerns subscales assessed perceived CRCI.^25^ The Cognitive Abilities subscales assessed CRCI by responding to statements like, “My memory has been as good as usual” and “I have been able to concentrate.” Higher scores indicate higher cognitive ability. The Cognitive Concerns scale has negatively worded items like, “My thinking has been slow” or “I have had trouble shifting back and forth between different activities that require thinking.” These subscales were rated using a 5‐point scale from “not at all” to “very much,” and total scores were sums from each items.^26^

### 2.3 Objective CRCI assessment

The objective CRCI assessment was done remotely using CANTAB^®^ (Cambridge Cognition Ltd., Cambridge, United Kingdom), a reliable and valid program used in several clinical populations, including cancer survivors, to assess cognitive performance.^27^ The specific CANTAB Cambridge Cognition^®^ cognitive domains were selected as they are common areas of concern for cancer survivors. For this study, we assessed the following cognitive function domains using specific CANTAB, Cambridge Cognition^®^ programs: (1) visuospatial working memory capacity using the CANTAB^®^ – spatial span (SSPFSL); (2) visual episodic memory and new learning using the CANTAB^®^ paired associates learning (PALTEA); (3) working memory and executive function via CANTAB^®^ – spatial working memory (SWMBE468); and (4) sustained attention via CANTAB^®^ – rapid visual information processing (RVPA).^6^

### 2.4 Assessment of confounders of cognitive function

Known confounders of cognitive function such as fatigue,^28^ depression,^29^ anxiety,^29^ and sleep disturbance^30^ were assessed using the 8‐item PROMIS^®^ short forms, all are standardized, reliable, and valid measures across populations, asking participants to recall their symptoms within the past week. Responses were rated using a 5‐point scale. Higher scores indicate worse symptomology.

### 2.5 *BDNF* rs6265 detection

*BDNF* rs6265 genotyping was done by collecting buccal swab samples using an OmniSwab kit (Qiagen, Maryland, US). Step‐by‐step instruction sheets for buccal swab collection, reminder emails to complete collection, and phone support from the research team related to the sample collection were sent to the study participants together with the OmniSwab kit. Upon receipt of the samples, they were deidentified and assigned unique ID numbers, stored at −80°C, and then batch‐shipped in ice to Genewiz (Azenta Life Sciences, New Jersey, US) for processing. The detailed methods to detect rs6265 genotypes were found in our previous paper.^24^, ^31^ In summary, DNA was extracted from each sample using the QIAamp^®^ DNA Mini Kit (QIAGEN, Maryland, US), and then a polymerase chain reaction (PCR) experiment was performed to analyze the area containing the *BDNF* rs6265 SNP (GenBank dbSNP: rs6265). The primer sequences were forward 5’–AGAAGAGGAGGCTCCAAAGG–3′ and reverse 5’–ACAAGGTGGCTTGGCCTAC–3′. The resulting DNA was purified enzymatically and the BigDye™ Terminator Cycle Sequencing Kit (ThermoFisher Scientific, Waltham, Massachusetts, US) was used for sequencing. For SNP detection, a threshold of 10% was set, and polymorphisms were identified based on the reference sequence, whenever possible, taking into account sequence quality and coverage. Data analysis was conducted using the DNASTAR Lasergene12^®^ software (DNASTAR, Inc., Madison, WI, US).^24^, ^31^

### 2.6 Statistical analyses

Descriptive statistics were employed to summarize the data, including the means and standard deviations (SD) for continuous variables that were normally distributed, median and interquartile ranges (IQR) for continuous variables that were not normally distributed, and numbers and percentages for categorical variables. To compare the cognitive function scores among the genotype groups, either one‐way analysis of variance (ANOVA) or the Kruskal–Wallis (KW) test was utilized, depending on the appropriateness. Subsequently, post hoc tests (e.g., two‐sample *t*‐tests or Wilcoxon rank‐sum tests) were done for pairwise comparisons. As a hypothesis‐generating analysis of the data collected from the parent study, a power analysis was not performed for this sub‐analysis. Statistical significance was defined with a *p*‐value < 0.05, and we adopted raw *p*‐values without adjustments for the post hoc multiple pairwise comparisons to avoid the loss of clinically meaningful results.

For cognitive outcomes that showed a genotype difference (with a *p*‐value less than 0.1 from ANOVA or KW test), multivariable regression analyses explored the effects of age and other confounders (fatigue, anxiety, depression, sleep disturbance) on the outcome variables in respect to the *BDNF* rs6265 genotypes. After assessing multicollinearity among the potential explanatory variables, age and the confounders listed above were entered into the model as co‐explanatory variables.^3^, ^4^ Entered co‐explanatory variables and final models for each outcome were determined based on the drop in dispersion test and *R* ^2^ values for each model. Statistical significance was defined with a *p*‐value < 0.05. Since the assumptions of the linear regression analysis did not hold for the selected cognitive function scores, rank‐based regression was used to analyze the data using the Rfit package.^32^ All analyses were conducted using R (version 4.2.1).

## 3 RESULTS

### 3.1 Sample description

A total of 357 female BCS were included in this secondary analysis. However, one participant had missing *BDNF* genotypic information, so was excluded; hence, the final analysis only included 356 study participants. Table 1 described the study participants' demographic and clinical profiles. Briefly, the mean (SD) age was 55.6 (9.8) years old, the median (IQR) years since cancer diagnosis were 4.0 (6.0), and 331 (92.7%) were self‐described as White. Of the 356 study participants, 232 (65.1%) had Val/Val, 112 (31.5%) had Val/Met, and 12 (3.4%) had Met/Met genotypes; of the total alleles, 576 (80.9%) were Val and the remaining 136 (19.1%) were Met (Table 2), which is equivalent to a published normative value.^33^ Hardy–Weinberg Equilibrium (*p* = 0.86) indicates that the observed genotype frequencies of the *BDNF* gene in the entire sample conform to the expectations of a homogenous population. These distributions are consistent with the expected *BDNF* genotype distribution of an overwhelmingly homogenous sample (93% White).^34^

**TABLE 1:** Differences in demographic characteristics among the *BDNF* genotypes (*n* = 356).

		Val/Val (*n* = 232)	Val/Met (*n* = 112)	Met/Met (*n* = 12)	*p*‐Value	Test
Age (years), mean (SD)		55.7 (9.67)	55.5 (10.2)	54.4 (9.60)	0.90	a
Years Since Cancer Diagnosis (years), median (IQR)		4 (6)	4 (5)	3.5 (7.5)	0.38	k
Race, number (%)	American Indian or Alaskan Native	2 (1)	0 (0)	0 (0)	<0.001	f
	Asian	0 (0)	1 (1)	1 (8)		
	Black	12 (9)	0 (0)	0 (0)		
	More than one race	4 (3)	1 (1)	0 (0)		
	Other	2 (1)	0 (0)	1 (8)		
	Unknown/Prefer not to answer	0 (0)	0 (0)	1 (8)		
	White	212 (86)	110 (98)	9 (75)		
Marital Status, number (%)	Divorced	32 (14)	9 (8)	1 (8)	0.02	f
	Living with partner	11 (5)	3 (3)	2 (17)		
	Married	161 (69)	79 (71)	4 (33)		
	Other/Prefer not to answer	4 (2)	3 (3)	0 (0)		
	Single	18 (8)	11 (10)	4 (33)		
	Widowed	6 (3)	7 (6)	1 (8)		
Education, number (%)	Associate's degree/some college	52 (22)	24 (21)	5 (42)	0.77	f
	High school graduate	11 (5)	7 (6)	1 (8)		
	Master's degree or equivalent	62 (27)	33 (29)	2 (17)		
	PhD or equivalent	24 (10)	8 (7)	1 (8)		
	Undergraduate/Bachelor's degree or equivalent	83 (36)	40 (36)	3 (25)		
Cancer Stage, number (%)	I	77 (33)	43 (38)	1 (8)	<0.001	f
	II	113 (49)	44 (39)	3 (25)		
	III	42 (18)	25 (22)	8 (67)		
Chemotherapy, number (%)	Yes	204 (88)	100 (89)	12 (100)	0.59	f
Surgery, number (%)	Yes	231 (99)	112 (100)	12 (100)	1	f
Radiation, number (%)	Yes	167 (72)	74 (66)	12 (100)	0.03	f

Abbreviations: a, analysis of variance; f, Fisher's exact test; IQR, interquartile range; k, Kruskal–Wallis test; SD, standard deviation.

**TABLE 2:** Frequencies of the *BDNF* rs6265 polymorphism genotypes and alleles (*n* = 356).

Genotype	Absolute frequency	Relative frequency (%)
Val/Val	232	65.1
Val/Met	112	31.5
Met/Met	12	3.4
Val allele	576	80.9
Met allele	136	19.1

### 3.2 Relationships between the *BDNF* rs6265 genotypes and cognitive functions

**FIGURE 1:**
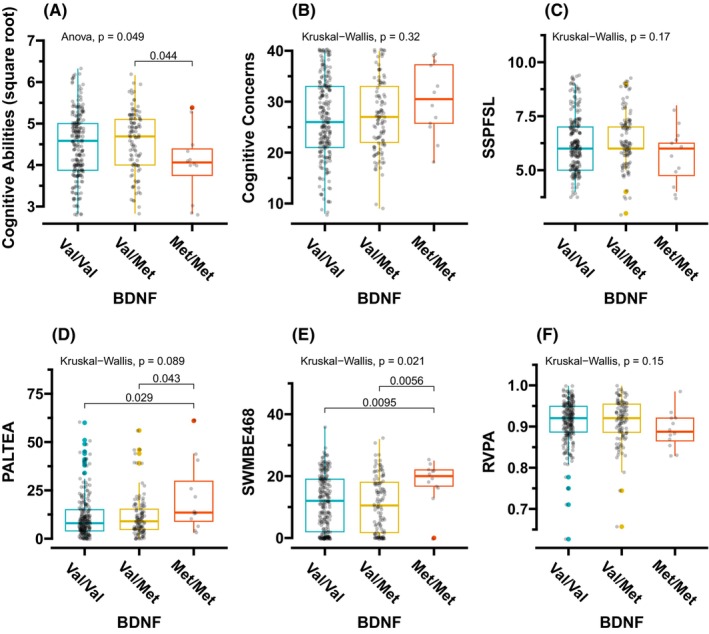
Differences in each cognitive function score per *BDNF* genotype. (A) Perceived cognitive abilities as assessed by PROMIS^®^ Cognitive Abilities. Higher scores indicate better cognitive ability. (B) Perceived cognitive concerns as assessed by PROMIS Cognitive Concerns. Higher scores indicate more cognitive concerns. (C) Visuospatial working memory capacity using the CANTAB‐spatial span (SSPFSL). Higher scores indicate better cognitive performance. (D) Visual episodic memory and new learning using the CANTAB‐paired associates learning (PALTEA). Higher scores indicate worse performance. (E) Working memory and executive function via CANTAB‐spatial working memory (SWMBE468). Higher scores indicate worse performance. (F) Sustained attention via CANTAB‐rapid visual information processing (RVPA). Higher scores indicate better cognitive performance. The one‐way analysis of variance or the Kruskal–Wallis tests followed by post hoc multiple comparisons using *t*‐test or Wilcoxon's rank sum test without *p*‐value adjustment was applied.

Figure 1 shows the intra‐genotype comparisons for each cognitive function domain score. PROMIS^®^ Cognitive Abilities scores were significantly different between the genotypes (ANOVA, *p* = 0.049), where it was significantly lower (worse) in the Met/Met genotype than Val/Met genotype (*p* = 0.044) with post hoc pairwise comparisons (Figure 1A). Participants with the Met/Met genotype had higher (worse) visual episodic memory and new learning as assessed by PALTEA compared to those with the Val/Val (*p* = 0.029) and the Val/Met (*p* = 0.043) genotypes (Figure 1D). Further, working memory and executive function as assessed by SWMBE468 were also higher (worse) for those with the Met/Met than those with the Val/Val (*p* = 0.01) and the Val/Met (*p* = 0.007) genotypes (Figure 1E).

While chemotherapy represents a significant risk factor for CRCI,^35^ we did not find a statistical difference in the six areas of cognitive function in our cohort. This is probably because 89% of the participants underwent chemotherapy, or we recruited those who had self‐reported CRCI. However, our investigation focused on examining the associations between *BDNF* rs6265 genotypes and cognitive function scores only in participants who received chemotherapy (as a sub‐group analysis, Figure S1) to avoid missing a clinically relevant result. For those who received chemotherapy, 204 participants (88%) had the Val/Val genotype, 100 participants (89%) with the Val/Met genotype, and 12 participants (100%) with the Met/Met genotype (Table 1). In this sub‐group analysis, the Met/Met genotype participants showed ‘worst visual episodic memory and new learning’ as assessed by PALTEA and ‘working memory and executive function’ as assessed by SWMBE468 (Figure S1D,E), replicating what was found in the analysis that included all participants (Figure 1D,E). In addition, participants with the Met/Met genotype had worse sustained attention as assessed by RVPA than those with the Val/Met genotype within this sub‐group of samples (Figure S1F).

### 3.3 Exploratory regression analysis using the rank regression model

Based on the intra‐genotype comparisons of the cognitive function scores that showed significant differences, PROMIS^®^ Cognitive Abilities (perceived cognitive function), PALTEA (visual episodic memory and new learning), and SWMBE468 (working memory, executive function) were selected as response variables for the multivariable regression analyses using the rank regression model (Table 3). In the final model, perceived cognitive impairment as assessed by PROMIS^®^ Cognitive Abilities was associated with age (slope = 0.09, *p* = 0.01) and anxiety (slope = −0.46, *p* < 0.001). Visual episodic memory and new learning as measured by PALTEA were associated with the *BDNF* rs6265 Met/Met genotype (slope = 7.25, *p* < 0.001), age (slope = 0.21, *p* < 0.001), and depression (slope = 0.12, *p* = 0.02). Working memory and executive function as assessed by SWMBE468 were associated with the *BDNF* rs6265 Met/Met genotype (slope = 6.92, *p* = 0.01), age (slope = 0.35, *p* < 0.001), and depression (slope = 0.19, *p* = 0.003).

**TABLE 3:** Multivariable rank regression models for all study participants.

	Coefficients	SE	*p‐*Value
Cognitive Abilities			
*BDNF* Val/Met	1.32	0.72	0.07
*BDNF* Met/Met	−2.87	1.85	0.12
Age	0.09	0.04	**0.01**
Anxiety	−0.46	0.05	**<0.001**
PALTEA			
*BDNF* Val/Met	0.53	0.84	0.53
*BDNF* Met/Met	7.25	2.18	**<0.001**
Age	0.21	0.04	**<0.001**
Depression	0.12	0.05	**0.02**
SWMBE468			
*BDNF* Val/Met	−0.65	0.99	0.51
*BDNF* Met/Met	6.92	2.56	**0.01**
Age	0.35	0.05	**<0.001**
Depression	0.19	0.06	**0.003**

*Note*: Rank‐based regression was used to analyze these data. Cognitive Ability: higher scores indicate better cognitive ability. PALTEA: higher scores indicate worse performance. SWMBE468: higher scores indicate worse performance.

As we did in the intra‐genotype comparisons, we also tested the multivariable rank regression models for those who received chemotherapy (Table S1). Perceived cognitive impairment as assessed by PROMIS^®^ Cognitive Abilities was associated with anxiety (slope = −0.47, *p* < 0.001). ‘Visual episodic memory and new learning’ as measured by PALTEA was associated with the *BDNF* rs6265 Met/Met genotype (slope = 6.95, *p* = 0.002) and age (slope = 0.24, *p* < 0.001). ‘Working memory and executive function’ as assessed by SWMBE468 was associated with the *BDNF* rs6265 Met/Met genotype (slope = 6.71, *p* = 0.01), age (slope = 0.32, *p* < 0.001), and depression (slope = 0.20, *p* = 0.002).

## 4 DISCUSSION

This study examined the relationships of *BDNF* rs6265 polymorphism with cognitive function domains as assessed by subjective questionnaires and the computerized CANTAB^®^ tasks in 356 female BCS. We found that the *BDNF* rs6265 Met/Met genotype contributed to worse visual episodic memory and new learning (as assessed by PALTEA) and working memory and executive function (as assessed by SWMBE486). These results suggest that female BCS carrying the *BDNF* rs6265 Met/Met genotype are more susceptible to cognitive dysfunction, especially learning, memory, and executive function, which are also affected by chemotherapy.^36^ These results were also confirmed in a sub‐group of the study participants who underwent chemotherapy, which is one of the major causes of CRCI in BCS.^37^ The results from the sub‐group analysis suggested that sustained attention as assessed by RVPA was also negatively affected by the Met/Met genotype. The study findings suggest that *BDNF* rs6265 polymorphisms may affect CRCI independently, separate from receiving chemotherapy; however, the interactions of the rs6265 polymorphism with chemotherapy should be investigated in a larger cohort.

The role of BDNF in brain synapse plasticity suggests its importance in learning and memory processes, guiding short‐term electrical changes as well as long‐term structural changes in synapses.^11^ One study investigating the relationship between BDNF expression and synaptic plasticity particularly long‐term potentiation, found that long‐term potentiation was impaired in *BDNF*‐mutant mice.^38^ Some studies have reported that the *BDNF* rs6265 Met allele is linked with poor episodic memory and abnormal hippocampal activity.^13^, ^39^ This evidence further strengthens the suggestion that cognitive dysfunction is influenced by the *BDNF* rs6265 polymorphism, as our findings showed that carrying the *BDNF* rs6265 Met/Met genotype was linked with learning and short‐term memory impairment. We previously reported lower learning and memory function among BCS compared to age‐ and education‐matched healthy controls.^40^ Our current findings also suggest that visual episodic memory and new learning may worsen in BCS carrying the Met/Met genotype of the *BDNF* rs6265 with age, although the slope for age was much smaller than that of the Met/Met genotype.

The *BDNF* rs6265 Met/Met genotype was negatively linked with visuospatial working memory capacity with respect to age and depression scores. Previous studies reported that older adults with the Met allele have better cognitive ability.^19^, ^20^, ^21^, ^22^ Thus, interventional strategies may need to be age‐appropriate. A longitudinal study with a sufficient sample size for the Met/Met genotype may reveal specific effects of age. Additionally, a sufficiently powered longitudinal study may establish a causal relationship between worsened visuospatial working memory capacity and depression; such a study may be useful to establish new preventive or treatment strategies. A previous review reported a negative relationship between blood BDNF and symptom severity in patients with major depression,^41^ which is a potential parameter explaining the relationship between cognitive impairment and depression.

A systematic review reported inconsistencies in the associations of *BDNF* rs6265 and cognitive performance.^23^ Of the 6 studies that explored the associations of *BDNF* rs6265 and cognitive performance in BCS, one study reported a protective effect of *BDNF* rs6265 against cognitive decline,^42^ while the other studies reported no association between the SNP and cognitive performance. Since some of the studies only observed the Met allele but not each genotype separately,^43^, ^44^, ^45^ they do not contradict our findings as our study observed a negative effect of the SNP on cognitive function in this population, specifically those with the Met/Met genotype but not with the Met allele. However, we still see the variability in the association of *BDNF* rs6265 and CRCI, which contributes to the evidence needed to advance our discussion on this topic. Several limitations of some studies in this review, as well as of our study, related to small sample sizes and the use of different objective cognitive performance tests remain a common challenge especially when comparing results between studies. However, our study has new information worth exploring further, particularly the worsening effect of the genotype on objectively assessed cognitive tasks in visual episodic memory, new learning, and visuospatial working memory capacity domains.

Another notable finding of this study from the regression analysis was that the depression score was associated with the two objective CRCI assessments. One systematic review and a meta‐analysis revealed that executive function, memory, and attention worsened in depressed patients.^46^ Another study reported that depressed older adults had a lower cognitive function, particularly in memory, executive function, and processing speed, and had lower serum BDNF concentrations than those without depressive symptoms.^47^ Previous studies reported that Met allele carriage, whether homozygous or heterozygous, is linked with lower blood BDNF levels.^48^, ^49^ While we did not quantify the BDNF levels in our cohort, our results suggest a potential mechanism for increased levels of BDNF in individuals with the Met allele: *BDNF* rs6265 Met/Met homozygotes, resulting in depressive symptom‐induced cognitive impairment in female BCS. However, this hypothesis needs to be further tested in a longitudinal study.

Our sampling method using social media and online cancer‐affiliated resource sites may introduce selection bias. We need to carefully consider the generalizability of our findings because those who were able to access the advertisement were more likely to participate in the current study. Approximately 93% of the participants in our study were White, which limited the interpretation of the results. Possessing the Met allele genotype of the *BDNF* rs6265 differs among populations, where Asians tend to more often carry this polymorphism (up to 72%) than other races.^50^ A previous review reported that while carrying the Met allele did not contribute to increasing Alzheimer's disease risk among Asian females, possessing the Met allele was found to contribute significantly to the increased risk of the disease in White females.^48^ In addition, racial differences in symptom reporting are well documented in patients with breast cancer.^51^ The contribution of race in understanding the role of the Met allele of *BDNF* rs6265 in altering cognitive function must be further explored to clarify the opposing results reported from our study and previous reports.^3^, ^4^, ^42^ We are currently widening our study enrollment to include a diverse group of participants from various ethnicities and racial categories to further validate the findings reported in this study.

Another limitation is that only 12 participants had the Met/Met genotype. Since the Met allele frequency was 19.1% in the current study (Table 2), it is equivalent to a published normative value,^33^ confirming adequate sample distribution used in this study to conduct this type of genetic analysis. The low Met/Met genotype frequency may have led to type II statistical errors. In fact, some results were not statistically significant, although they trended towards a statistical difference. This low Met/Met genotype frequency also led us to hesitate to perform detailed sub‐group analyses to examine some potential confounders. For example, our results may have been affected by race, marital status, clinical stages of cancer, and radiation as the proportion of each item in the variables varied among the *BDNF* genotypes (Table 1). However, we did not find significant differences in cognitive function (in any domain) between cancer stages or cancer patients previously subjected to radiation (not shown). However, the present findings will allow us to plan a more robust study design including a longitudinal approach capturing pre‐treatment cognitive performance testing to further advance our understanding of the mechanisms underpinning CRCI, as recommended by the International Cognition and Cancer Task Force.^45^ Further, intentional investigations using SNP and CRCI data from more diverse populations should be conducted to understand the existence of racial variations in the effects of *BDNF* genotypes on CRCI. The role of *BDNF* rs6265 polymorphism on cognitive function is crucial information to establish tailor‐made prevention and treatment strategies for CRCI in BCS.

## 5 CONCLUSIONS

We found a link between the *BDNF* rs6265 Met/Met genotype to CRCI in female BCS, particularly in visual episodic memory, working memory, new learning, and executive function. The observed polymorphism demonstrated independent effects on the cognitive function domains with age, chemotherapy, and depression in our BCS cohort. These results can be foundational and are much needed to identify specific cognitive function domains affected by this polymorphism in the clinical setting.

## AUTHOR CONTRIBUTIONS

**Taichi Goto:** Formal analysis (lead); writing – original draft (lead); writing – review and editing (equal). **Leorey N. Saligan:** Conceptualization (equal); resources (equal); writing – review and editing (equal). **Xiaobai Li:** Formal analysis (equal); writing – review and editing (equal). **Lichen Xiang:** Writing – review and editing (equal). **Catherine Kwiat:** Writing – original draft (supporting); writing – review and editing (equal). **Christopher Nguyen:** Writing – original draft (supporting); writing – review and editing (equal). **Adele Crouch:** Data curation (equal); writing – review and editing (supporting). **Diane Von Ah:** Conceptualization (equal); data curation (equal); methodology (equal); writing – review and editing (equal).

## FUNDING INFORMATION

The current study received financial support from Indiana University, the National Institutes of Health, National Institute of Nursing Research, Division of Intramural Research (NIH/NINR) and The Ohio State University College of Nursing and The Ohio State University Comprehensive Cancer Center. The content is solely the responsibility of the authors and does not necessarily represent the official views of NIH or NINR.

## CONFLICT OF INTEREST STATEMENT

The authors declare no conflicts of interest.

## INSTITUTIONAL REVIEW BOARD STATEMENT

Approval for this study was granted by both a Midwest comprehensive cancer center and the Institutional Review Board (IRB) of Indiana University, under Protocol number 2009676157.

## INFORMED CONSENT STATEMENT

Prior to administering any study outcomes, informed consents were obtained from all participants.

## Supporting information


Data S1.

## Data Availability

Data analysis of the parent study is ongoing. Requesting data for secondary analysis can be made by contacting the authors.
