# Supplementary material for: Associations of brain‐derived neurotrophic factor rs6265 polymorphism and cognitive function in breast cancer survivors from a cross‐sectional study
Source: Cancer Med. 2024 Feb 4;13(2):e6975. doi: 10.1002/cam4.6975 (PMC10839126; doi:10.1002/cam4.6975)
Supplement: Supplementary file 1 — Data S1. [file CAM4-13-e6975-s001.docx]

**Supplementary materials**

**Figure S1.** Differences in each cognitive function score per *BDNF* genotype in the study participants undergoing chemotherapy.

A) Perceived cognitive abilities as assessed by PROMIS Cognitive Abilities. Higher scores indicate better cognitive ability. B) Perceived cognitive concerns as assessed by PROMIS Cognitive Concerns. Higher scores indicate more cognitive concerns. C) Visuospatial working memory capacity using the CANTAB-spatial span (SSPFSL). Higher scores indicate better cognitive performance. D) Visual episodic memory and new learning using the CANTAB paired associates learning (PALTEA). Higher scores indicate worse performance. E) Working memory and executive function via CANTAB-spatial working memory (SWMBE468). Higher scores indicate worse performance. F) Sustained attention via CANTAB-rapid visual information processing (RVPA). Higher scores indicate better cognitive performance. The one-way analysis of variance or the Kruskal-Wallis tests followed by *post hoc* multiple comparisons using *t*-test or Wilcoxon’s rank sum test without *p*-value adjustment was applied.

| **Table S1.** Multivariable rank regression models for study participants with chemotherapy | | | | |
| --- | --- | --- | --- | --- |
|  |  | Coefficients | SE | *p-*value |
| Cognitive Abilities | |  |  |  |
|  | BDNF Val/Met | 1.31 | 0.76 | .09 |
|  | BDNF Met/Met | -2.86 | 1.85 | .12 |
|  | Age | 0.07 | 0.04 | .08 |
|  | Anxiety | -0.47 | 0.05 | **< .001** |
| PALTEA | |  |  |  |
|  | BDNF Val/Met | 0.29 | 0.92 | .76 |
|  | BDNF Met/Met | 6.95 | 2.23 | **.002** |
|  | Age | 0.24 | 0.04 | **< .001** |
| SWMBE468 | |  |  |  |
|  | BDNF Val/Met | -1.33 | 1.06 | .21 |
|  | BDNF Met/Met | 6.71 | 2.59 | **.01** |
|  | Age | 0.32 | 0.05 | **< .01** |
|  | Depression | 0.20 | 0.07 | **.003** |
| Rank-based regression was used to analyze these data. Cognitive Ability: higher scores indicate higher cognitive ability. PALTEA: higher scores indicate worse performance. SWMBE468: higher scores indicate worse performance. | | | | |
